# Structural retinal changes in cerebral small vessel disease

**DOI:** 10.1038/s41598-022-13312-z

**Published:** 2022-06-03

**Authors:** S. Magdalena Langner, Jan H. Terheyden, Clara F. Geerling, Christine Kindler, Vera C. W. Keil, Christopher A. Turski, Gabrielle N. Turski, Charlotte Behning, Maximilian W. M. Wintergerst, Gabor C. Petzold, Robert P. Finger

**Affiliations:** 1 Department of Ophthalmology, University Hospital Bonn, Ernst-Abbe-Str. 2, 53127 Bonn, Germany; 2 Department of Neurology, University Hospital Bonn, Bonn, Germany; 3 German Center for Neurodegenerative Diseases (DZNE), Bonn, Germany; 4 Department of Radiology, Amsterdam UMC, Location VUmc, De Boelelaan 1117, 1081 HV Amsterdam, The Netherlands; 5 Institute of Biomedical Statistics, Computer Science and Epidemiology, University Hospital Bonn, Bonn, Germany; 6 Division of Vascular Neurology, University Hospital Bonn, Bonn, Germany

**Keywords:** Cerebrovascular disorders, Diagnostic markers

## Abstract

Cerebral small vessel disease (CSVD) is an important contributor to cognitive impairment and stroke. Previous research has suggested associations with alterations in single retinal layers. We have assessed changes of all individual retinal layers in CSVD using high-resolution optical coherence tomography (OCT) for the first time. Subjects with recent magnetic resonance imaging (MRI) underwent macular and peripapillary retinal imaging using OCT for this case–control study. Number and volume ratio index (WMRI) of white matter lesions (WML) were determined on MRI. Data were analyzed using multiple linear regression models. 27 CSVD patients and 9 control participants were included. Ganglion cell layer (GCL) volume was significantly reduced in patients with CSVD compared to age-matched controls (*p* = 0.008). In patients with CSVD, larger foveal outer plexiform layer (OPL) volume and decreased temporal peripapillary retinal nerve fiber layer (RNFL) thickness were significantly associated with a higher WMRI in linear regression when controlling for age (*p* ≤ 0.033). Decreased foveal GCL volume and temporal-inferior RNFL thickness at Bruch’s membrane opening (MRW), and increased temporal MRW were associated with a higher WML burden (*p* ≤ 0.037). Thus, we identified alterations in several OCT layers in individuals with CSVD (GCL, OPL, MRW and RNFL). Their potential diagnostic value merits further study.

## Introduction

Cerebral small vessel disease (CSVD) is associated with hypertension, hypercholesterolemia and obesity and a risk factor for stroke, vascular cognitive impairment and depression, thus contributing considerably to global mortality^1–3^. It is characterized by narrowing of the vessel lumen and failure of vascular autoregulation^3^. CSVD can be clinically silent, but is commonly associated with a decline in cognitive function with disease progression^4–6^. The lifetime probability to develop CSVD is substantial. For example, only 8% of the elderly general population in the Rotterdam study had no magnetic resonance imaging (MRI) changes suggestive of CSVD^2^. Prevalence data from other sources are similarly high^7^.

Cerebral small vessels are difficult to assess using even high-resolution MRI. Thus, diagnostic markers used are downstream cerebral changes caused by the resulting ischemia including periventricular and subcortical white matter lesions (WML) which can be detected and quantified on MRI^8,9^. Further diagnostic procedures include neuropsychological and neurological tests, doppler-sonography and computer tomography which are primarily used to evaluate the clinical impact of CSVD^6,8,10^. In addition, several contraindications preclude MRI in a considerable number of patients.

The retina is much easier accessible for imaging and allows for the non-invasive, high-resolution assessment of neuronal tissue using e.g. optical coherence tomography (OCT)^11,12^. Given the common embryological origin and the common blood supply of both organs through the internal carotid artery, one might expect changes in the retina in CSVD and in fact, peripapillary retinal nerve fiber layer (pRNFL) thickness was found to be decreased in patients with a hereditary form of CSVD^13,14^. Furthermore, defects of the RNFL appeared to be associated with WML on fundus photographs^15^.

Therefore, we here assessed the association of WML on MRI with structural OCT parameters and provide information on the extend and characteristics of macular and optic disc changes in all retinal layers in CSVD to identify markers, which might help to detect this disease at an earlier stage.

## Results

36 eyes of 36 participants (27 participants with CSVD and 9 controls) were included in the analysis (Table 1). After screening of 62 individuals initially, 26 subjects were excluded due to retinal pathology (n = 18 subjects), age (n = 7 controls could not be age-matched within 5 years to patients), cerebral WML other than CSVD (n = 1) and poor OCT image quality (n = 2 scans). 19% of our participants were classified as cognitively impaired. 14 participants (39%) were imaged with a 1.5 T MRI device and 22 participants (61%) with 3 T MRI device (Table 1).

**Table 1:** Demographic variables and imaging parameters of patients and controls.

	Total	CSVD	controls	Age matched CSVD	*P*1*	*P*2*
Gender (m/f)	15/21	12/15	3/6	4/5	0.641	0.730
MRI (1.5 T/3 T)	22/14	17/10	5/4	6/3	0.747	0.730
Fazekas (score 0/1/2/3)	9/14/10/3	0/14/10/3	9/0/0/0	0/5/4/0		
Fazekas (median; IQR)	1; 2	1; 1	0;0	1; 1	**< 0.001**	**< 0.001**
Age (years)	63 ± 10	65 ± 11	57 ± 5	57 ± 4	0.073	1.000
MoCA	25 ± 4	25 ± 4	25 ± 3	26 ± 3	0.971	0.436
Cognitively impaired^49^	19%	22%	11%	11%		
WMRI	0.408 ± 0.663	0.536 ± 0.724	0.025 ± 0.027	0.232 ± 0.292	**< 0.001**	**0.006**
NOL	14 ± 9	17 ± 9	6 ± 6	13 ± 9	**< 0.001**	**0.031**
Total retina volume (mm^3^)	2.32 ± 0.01	2.31 ± 0.11	2.36 ± 0.07	2.31 ± 0.08	0.101	0.136

Values are mean ± SD and the volume of the retina corresponds to the foveal and parafoveal volumes (3-mm-circle). *Mann–Whitney-U-test was performed to compare the groups. P1, comparison between all patients and controls; P2, comparison between age matched patients and control. Significant results (*p* < 0.05) are displayed in bold.

CSVD, patients with cerebral small vessel disease; MRI, device used for MRI imaging; 1.5 T, Philips Ingenia 1.5 T; 3 T, Achieva TX 3 T, m, male; f, female; Fazekas, Fazekas score; IQR, interquartile range; MoCA, Montreal Cognitive Assessment; WMRI, white matter lesions volume ratio index; NOL, number of white matter lesions.

As expected, patients with CSVD had significantly more WML (*p* < 0.001) and higher WML volume ratio index (WMRI) (*p* < 0.0001) compared to control participants. However, the patient group was noticeably older than the control group which is why we performed an age-matched subgroup analysis (see below). The ganglion cell layer (GCL) volume was significantly lower in patients with CSVD compared to controls (0.31 ± 0.04 versus 0.34 ± 0.02, *p* = 0.032). Neither the other OCT parameters nor the Montreal Cognitive Assessment (MoCA) score were significantly different between patients and controls. In the receiver operating characteristic (ROC) analysis, the GCL volume was significantly associated with CSVD disease status (AUC 0.743; 95%-CI 0.578, 0.908; after adjusting for age: AUC 0.790; 95%-CI 0.641, 0.939). The MoCA-score was not associated with presence of CSVD (AUC 0.506; 95%-CI, 0.302, 0.701).

### Age-matched cases and controls

In a subgroup analysis of age-matched CSVD cases (Fazekas > 0) with controls (Fazekas = 0; see Table 1) we found that GCL volume was significantly reduced in patients with CSVD compared to age-matched controls (Table 2). In the analysis of the individual retinal sectors, the parafoveal nasal, inferior and superior volumes of the GCL were significantly reduced in CSVD compared to controls (Table 2).

**Table 2:** Comparison of structural retinal parameters between patients with cerebral small vessel disease and age-matched controls.

Parameter	Controls	CSVD	P*
**GCL (mm**^**3**^**)**	**0.34 ± 0.02**	**0.30 ± 0.02**	**0.008**
Foveal	0.01 ± 0.00	0.01 ± 0.00	0.730
**Pf. nasal**	**0.08 ± 0.01**	**0.07 ± 0.00**	**0.014**
**Pf. inferior**	**0.08 ± 0.01**	**0.07 ± 0.01**	**0.019**
Pf. temporal	0.08 ± 0.00	0.07 ± 0.01	0.063
**Pf. superior**	**0.08 ± 0.00**	**0.07 ± 0.01**	**0.011**
IPL (mm^3^)	0.28 ± 0.02	0.27 ± 0.02	0.094
INL (mm^3^)	0.27 ± 0.02	0.27 ± 0.02	1.00
OPL (mm^3^)	0.22 ± 0.06	0.20 ± 0.02	0.340
ONL (mm^3^)	0.51 ± 0.06	0.55 ± 0.03	0.094
RPE (mm^3^)	0.11 ± 0.02	0.10 ± 0.02	0.387
pRNFL (µm)	75 ± 6	72 ± 4	0.222
MRW (µm)	341 ± 66	333 ± 65	0.796

Values are mean ± SD and the volumes of the macular layers correspond to the foveal and parafoveal volumes (3-mm-circle). *Mann–Whitney-U-test was performed to compare the two groups. Significant results (*p* < 0.05) are displayed in bold.

CSVD, patients with cerebral small vessel disease; GCL, ganglion cell layer; pf., parafoveal; IPL, inner plexiform layer; INL, inner nuclear layer; OPL, outer plexiform layer; ONL, outer nuclear layer; RPE, retinal pigment epithelium; pRNFL, peripapillary retinal nerve fiber layer; MRW, Bruch’s membrane opening-minimum rim width.

### Associations of MRI biomarkers with OCT parameters

In a subgroup analysis of CSVD cases only, the WMRI was associated with an increase in volume of the foveal outer plexiform layer (OPL). A decrease of the inner plexiform layer (IPL), the pRNFL and Bruch’s membrane opening-minimum rim width (MRW) were significantly associated with a higher WMRI (Table 3 and Supplementary Table 1). After adjusting for age, an increase in the foveal OPL and a decrease in the peripapillary MRW and pRNFL were significantly associated with a higher WMRI (Table 3 and Supplementary Table 1). In age-adjusted multivariable linear regression using backward elimination, foveal OPL volume (β = 81.799; 95%-CI 21.927, 141.671; *p* = 0.01), temporal pRNFL thickness (β = − 0.021; 95%-CI − 0.039, − 0.002; *p* = 0.033) and age (β = 0.032; 95%-CI 0.017, 0.047; *p* < 0.001) remained associated with WMRI.

**Table 3:** Results of linear regression of structural retinal parameters and white matter lesions volume ratio index in patients with cerebral small vessel disease.

Structural retinal parameter	WMRI association		WMRI association, age adjusted	
	β [95%-CI]	*p*	β [95%-CI]	*p*
**IPL**				
Pf. nasal	**− 45.261 [− 86.436, − 4.085]**	**0.033**	**− **27.364 [**− **61.017, 6.289]	0.106
Pf. inferior	**− 31.918 [− 59.769, − 4.068]**	**0.026**	**− **21.373 [**− **43.621, 0.874]	0.059
**OPL**				
Foveal	**137.950 [62.956, 212.944]**	**0.001**	**101.841 [40.874, 162.808]**	**0.002**
**pRNFL**				
Temporal	**− 0.032 [− 0.060, − 0.005]**	**0.024**	**− 0.029 [− 0.049, − 0.009]**	**0.007**
PMB	**− 0.038 [− 0.075, − 0.001]**	**0.042**	**− 0.039 [− 0.064, − 0.013]**	**0.005**
**MRW**				
Nasal	**− 0.003 [− 0.007, 0.000]**	**0.044**	**− **0.002 [**− **0.005, 0.001]	0.234
Nasal inferior	**− 0.004 [− 0.007, 0.000]**	**0.030**	**− 0.003 [− 0.005, 0.000]**	**0.024**
Temporal inferior	**− 0.004 [− 0.008, − 0.001]**	**0.017**	**− 0.003 [− 0.006, − 0.001]**	**0.016**
PMB	**− 0.004 [− 0.007, 0.000]**	**0.033**	**− **0.002 [**− **0.005, 0.001]	0.209
Nasal superior	**− 0.004 [− 0.007, − 0.001]**	**0.018**	**− **0.002 [**− **0.005, 0.000]	0.087

Associations between retinal parameters and WMRI were assessed using linear regression. Model 1: univariate regression, Model 2: multivariable regression adjusted for age. Significant associations (*p* < 0.05) are displayed in bold.

In this table, only parameters are listed with a *p* value < 0.05 in at least one linear regression model. Further results are listed in the supplement. The volumes of the macular layers correspond to the foveal and parafoveal volumes (3-mm-circle).

WMRI, white matter lesions volume ratio index; β, standardized coefficient; CI, confidence interval; IPL, inner plexiform layer; pf., parafoveal; OPL, outer plexiform layer; pRNFL, peripapillary retinal nerve fiber layer; PMB, papillo-macular bundle; MRW, Bruch’s membrane opening-minimum rim width.

Most of the peripapillary MRW-locations and both peripapillary (pRNFL) and macular layers (IPL, outer nuclear layer, OPL) were significantly associated with the number of WML (NOL) (Table 4 and Supplementary Table 2). After adjusting for age, GCL, IPL and OPL volumes as well as MRW and pRNFL thickness were significantly associated with NOL (Table 4 and Supplementary Table 2). In stepwise multivariate linear regression foveal GCL volume (β = − 1266.561; 95%-CI − 1901.908, − 631.215; *p* < 0.001), temporal inferior MRW (β = − 0.111; 95%-CI − 0.159, − 0.062; *p* < 0.001), temporal MRW (β = 0.057; 95%-CI 0.004, 0.109; *p* = 0.037) and age (β = 0.547; 95%-CI 0.349, 0.746; *p* < 0.001) remained associated with NOL.

**Table 4:** Results of linear regression of structural retinal parameters and number of white matter lesions in patients with cerebral small vessel disease.

Structural retinal parameter	NOL association		NOL association, age adjusted	
	β [95%-CI]	*p*	β [95%-CI]	*p*
**Total retina**				
Pf. superior	**− 159.270 [− 316.458, − 2.082]**	**0.047**	− 99.825 [− 243.197, 43.547]	0.164
**GCL**				
Total volume	− 72.052 [− 159.997, 15.894]	0.104	**− 72.245 [− 144.055, − 0.435]**	**0.049**
Foveal	− 591.667 [− 1671.649, 488.315]	0.270	**− 874.843 [− 1743.724, − 5.961]**	**0.049**
Pf. nasal	− 269.435 [− 635.884, 97.014]	0.142	**− 306.899 [− 602.325, − 11.473]**	**0.042**
**IPL**				
Pf. nasal	**− 693.590 [− 1237.220, − 149.959]**	**0.014**	**− 500.646 [− 993.331, − 7.961]**	**0.047**
Pf. inferior	**− 452.976 [− 827.535, − 78.417]**	**0.020**	**− 336.222 [− 668.912, − 3.533]**	**0.048**
**OPL**				
Foveal	**1550.000 [442.428, 2657.572]**	**0.008**	**1129.675 [112.316, 2147.033]**	**0.031**
Pf. superior	**412.534[110.271, 714.797]**	**0.009**	268.754 [− 25.311, 562.819]	0.071
**ONL**				
Pf. superior	**− 325.122 [− 557.611, − 92.634]**	**0.008**	− 173.958 [− 433.211, 85.294]	0.179
**pRNFL**				
N/T	15.701 [− 2.940, 34.341]	0.095	**15.711 [0.737, 30.685]**	**0.041**
Temporal	**− 0.475 [− 0.853, − 0.097]**	**0.016**	**− 0.432 [− 0.736, − 0.128]**	**0.007**
PMB	− 0.481 [− 0.993, 0.030]	0.064	**− 0.487 [− 0.893, − 0.081]**	**0.021**
Temporal superior	**− 0.226 [− 0.443, − 0.008]**	**0.043**	**− 0.189 [− 0.371, − 0.008]**	**0.041**
**MRW**				
Total	**− 0.074 [− 0.128, − 0.020]**	**0.009**	**− 0.055 [− 0.103, − 0.007]**	**0.027**
Nasal	**− 0.068 [− 0.110, − 0.026]**	**0.003**	**− 0.050 [− 0.089, − 0.010]**	**0.016**
Nasal inferior	**− 0.067 [− 0.108, − 0.026]**	**0.002**	**− 0.059 [− 0.092, − 0.026]**	**0.001**
Temporal inferior	**− 0.076 [− 0.120, − 0.031]**	**0.002**	**− 0.065 [− 0.101, − 0.028]**	**0.001**
Temporal	**− 0.067 [− 0.116, − 0.019]**	**0.009**	**− 0.048 [− 0.092, − 0.003]**	**0.039**
PMB	**− 0.063 [− 0.107, − 0.018]**	**0.008**	− 0.042 [− 0.085, 0.000]	0.050
Temporal superior	**− 0.054 [− 0.105, − 0.004]**	**0.035**	− 0.039 [− 0.083, 0.006]	0.085
Nasal superior	**− 0.065 [− 0.109, − 0.020]**	**0.006**	**− 0.046 [− 0.087, − 0.005]**	**0.029**

Associations between retinal parameters and NOL were assessed using linear regression. Model 1: univariate regression, Model 2: multivariable regression adjusted for age; significant associations (*p* < 0.05) are displayed in bold.

In this table, only parameters are listed with a *p* value < 0.05 in at least one linear regression model. Further results are listed in the supplement. The volumes of the macular layers correspond to the foveal and parafoveal volumes (3-mm-circle).

NOL, number of white matter lesions; β, standardized coefficient; CI, confidence interval; pf., parafoveal; GCL, ganglion cell layer; IPL, inner plexiform layer; OPL, outer plexiform layer; ONL, outer nuclear layer; pRNFL, peripapillary retinal nerve fiber layer; N/T, ratio nasal to temporal; PMB, papillo-macular bundle; MRW, Bruch’s membrane opening-minimum rim width.

## Discussion

In this study we found that peripapillary and macular OCT parameters, most prominently the GCL, were associated with both the extent of CSVD lesions on MRI as well as reduced in comparison to controls without CSVD. These results support a relationship of retinal structural parameters and cerebral changes in CSVD and may be useful in the detection and staging of CSVD. Further studies are needed to better characterize this relationship.

Our findings are in agreement with published studies. Aggarwal et al. reported a significant reduction of ganglion cells in patients with chronic non‐arteritic anterior ischemic optic neuropathy^16^. This is consistent with the observation of GCL reduction in other diseases associated with ischemia like diabetes and retinal vein occlusion^17,18^. Vascular factors also play an important role in the pathogenesis of many neurodegenerative diseases such as multiple sclerosis, Alzheimer’s disease and Parkinson’s disease, in all of which a significant GCL difference between cases and controls has been described^19–29^. Nevertheless, the use of retinal GCL volume as a biomarker in CSVD needs to be investigated in further studies.

Interestingly, we found an OPL volume increase in association with a higher WMRI and a higher NOL. OPL thickening could be found as well in two studies which compared patients with Parkinson’s disease to controls^30,31^. Other studies could not confirm this finding^32,33^. In multiple sclerosis and Alzheimer’s disease, the OPL has been reported to be decreased or unchanged in patients compared to controls, whereas other layers like the inner and outer nuclear layer have been reported to be significantly thicker in patients with neurodegenerative diseases^34–36^. Explanations offered for increases in thickness or volume include local inflammation, ischemia and cellular movements^35^. Ischemia has been demonstrated to lead to a significantly thickened OPL in diabetic retinopathy^37^.

The absence of any difference in pRNFL thickness between patients and controls in our study is in contrast to two studies which investigated patients with Cerebral Autosomal Dominant Arteriopathy with Subcortical Infarcts and Leukoencephalopathy (CADASIL) and reported RNFL thickness to be reduced compared to controls^13,14^. Some of the included CADASIL-patients showed more severe symptoms such as migraine-like episodes with aphasia and hemiparesis^13^. This may explain our different results as patients and controls were largely clinically silent and not cognitively affected in our study. However our results on pRNFL measurements are in agreement with a recent study of Lee et al. on vascular changes in CSVD patients who did not find any differences between cases and controls^38^. In addition to Lee et al., we also investigated structural retinal parameters and found a significant association between the decrease of pRNFL thickness and an increase of WML. Similar results were obtained in a Korean study, which could demonstrate associations between white matter lesions and RNFL defects visible on fundus photographs in a large population^15^. Using MRW, which quantifies pRNFL thickness whilst taking local anatomic conditions (i.e. Bruch’s Membrane Opening, BMO) into account, we found significant associations with both WMRI and the NOL which were in keeping with expectations for pRNFL thickness based on published literature^36,39,40^. MRW is not widely used yet, but promises to be a useful parameter with theoretically better reproducibility compared to pRNFL thickness^41^.

The strengths of this study include the thorough screening for concurrent neurological or ocular comorbidities, the use of high-resolution MRI, a complete ophthalmic assessment, the use of state-of-the-art high-resolution SD-OCT imaging including novel read-outs corrected for ocular anatomy such as MRW and the use of standardized and published automated image analysis for both MRI and OCT. To date, this study is the first to investigate structural retinal changes considering all layers assessed by OCT in patients with CSVD after few studies assessed single-layer structural changes or structural and flow changes within the retinal vasculature^38,42–44^. The limitations of our study include foremost its relatively small sample size. As our study was designed as an exploratory study, confirmatory studies with larger sample sizes are needed. Different MRI devices and sequences were used in a clinical routine setting due to different medical indications for MRI imaging which could have an influence on the comparability and applicability to a population-based setting. In order to ensure comparability we used the LST- lesion prediction algorithm (LPA) segmentation method, which has proven good performances within and across scanners for white matter hyperintensity segmentation in a multicenter dataset^45^. Inaccuracies may have occurred due to the use of 2D instead of 3D data sets for the LST-LPA^46^. Nevertheless, the LST-LPA has already been demonstrated to be valid and reliable in 2D data sets^47^.

Non-neurological and non-ocular comorbidities were not assessed which might have affected our findings. One patient had a meningioma which had no influence on the evaluation of the Fazekas score or on the lesion segmentation and assumingly did not affect OCT measurements based on its localization. A further limitation is the time lag between MR-and OCT-imaging which might have led to an underestimation of effect size as cerebral CVSD lesions might have progressed in the meantime. We did not correct for multiple testing as this was an exploratory study, which might lead to spurious associations. Thus, any findings need confirmation in additional studies.

In conclusion, several retinal structural changes are present in CSVD patients, related to the extent of the cerebral changes and independent of their age. Many of these changes are unspecific in isolation but future studies may succeed in identifying a specific profile of retinal changes able to aid in the detection and monitoring of CSVD.

## Methods

### Participants

A total of 62 subjects (44 patients with CSVD and 18 control participants without CSVD or ocular diseases) were recruited from a database of the Department of Neurology at the University Hospital Bonn for this case–control study. All subjects had undergone cerebral MRI for clinical indications. The study followed the tenets of the Declaration of Helsinki and was approved by the Ethics Committee of the University Hospital Bonn (consecutive Number: 281/17). Written informed consent was obtained from all participants.

Potential participants were identified using an internal MRI database. The search algorithm included "MRI obtained between 01/01/2017 and 05/31/2019", as well as the keywords "headache," "dizziness," "syncope," "paresthesia," "exclusion of microembolization," and "exclusion of metastases".

Exclusion criteria were any eye diseases including glaucoma and retinal diseases, any opacity of the optical media interfering with retinal imaging, malignancy, major stroke, larger intracranial lesions or structural anomalies affecting the MRI analysis.

All participants underwent cognitive testing using the MoCA^48^. A result of 23 or more points was classified as cognitively normal^49^.

### MRI imaging

For the clinically indicated 1.5 or 3 T MRI-Imaging (Philips Ingenia 1.5 T and Achieva TX 3 T; Philips Healthcare, Best/The Netherlands) an 8-channel head coil was used.

Axial whole-brain 2D fluid-attenuated inversion recovery (FLAIR) images with a slice thickness of 6 mm, no gap and an echo time of 140 ms were evaluated for all patients. The FLAIR sequence of the 1.5 T MRI scanner had a matrix of 352 × 352, a repetition time of 11 s and an inversion time of 2800 ms. The corresponding sequence of the 3 T MRI scanner had a matrix of 512 × 512, a repetition time of 12 s and an inversion time of 2850 ms.

All patients and controls were independently evaluated and clinically diagnosed by two board certified neuroradiologists, based on the microvascular leukoencephalopathy score according to Fazekas et al.^50^ The score describes the quantity of white matter T2 hyperintense lesions. A score of 0 refers to the absence of periventricular or deep WML, a score of 1 to punctate foci or pencil thin lesions. The lesions form a smooth periventricular “halo” or become confluent at a score of 2. A Fazekas score of 3 describes large confluent and irregular lesion areas, which extend periventricular into the deep white matter.

**Figure 1:**
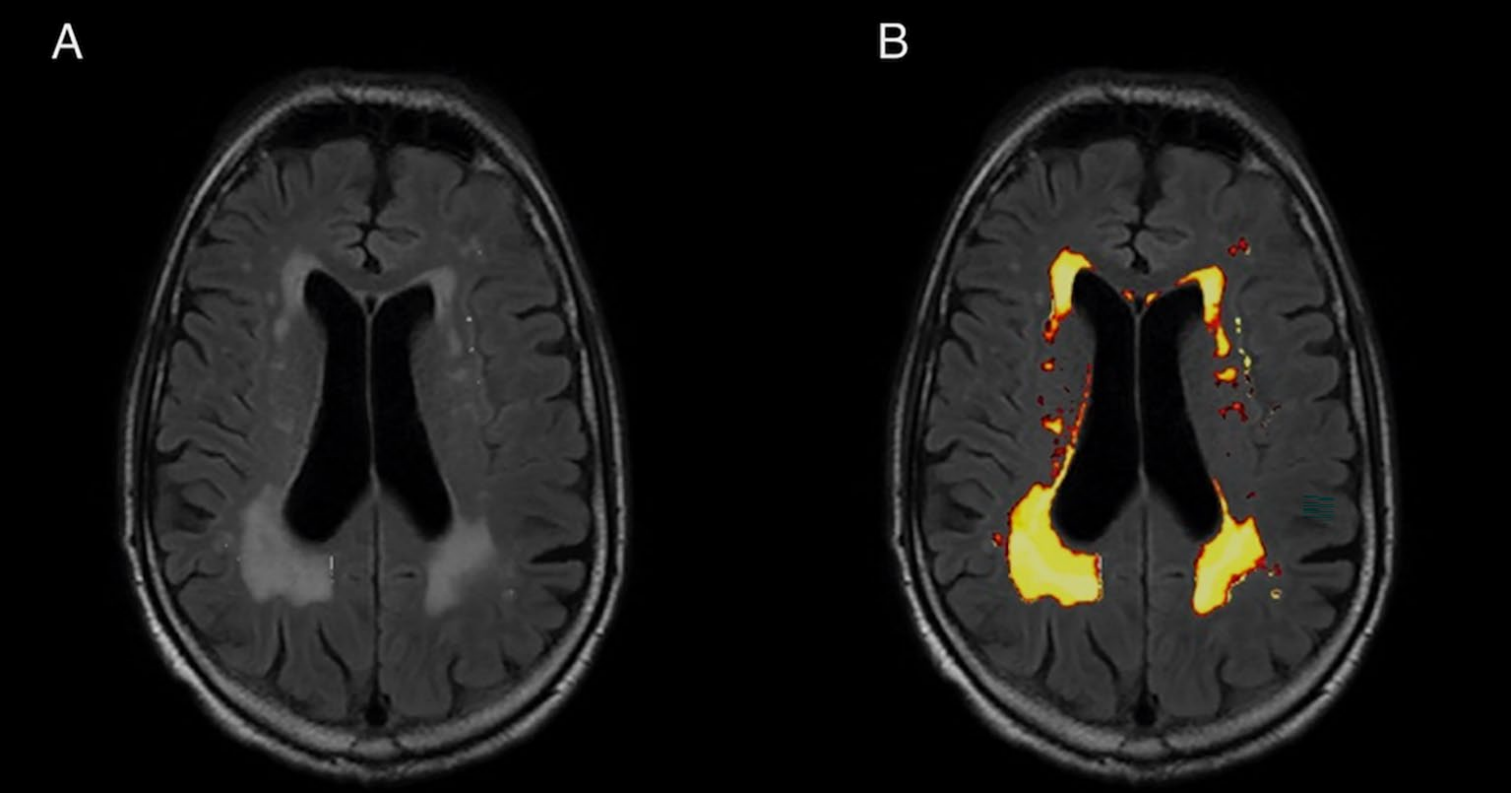
T2 FLAIR images from a 75-year old participant with hyperintense lesions (Fazekas 3) due to cerebral small vessel disease. (**A**) T2 FLAIR image showing multiple hyperintense white matter lesions. (**B**) Results of the white matter lesions segmentation using the lesion prediction algorithm. The white matter lesions detected by the algorithm are presented in red-yellow.

The lesions were automatically segmented with the LST Toolbox (version 2.0.15 for SPM12)^51^. LPA was used to calculate NOL and volume of the WML in the 2D data sets. The algorithm was developed for and trained on the segmentation of lesions in multiple sclerosis^52,53^. Further studies have already used the LST for other diseases like Amyotrophic Lateral Sclerosis and diabetes mellitus^54,55^. The algorithm only needs FLAIR sequences for the analysis. It consists of a binary classifier in the form of a logistic regression model and uses a lesion belief map and a spatial covariate, which assesses the changes in lesion probability for each voxel. In new images, the algorithm evaluates the voxel specific lesion probability, based on the previous parameters of the model, and uses the results for the lesion segmentation (Fig. 1)^53^. The automated segmentation of volume and NOL was manually checked for accuracy. Following this, the degree of lesions of each subject was related to the total intracranial volume (TIV). The 2D FLAIR images were also used to calculate the TIV. First, the LST toolbox was used to annotate the WML. The subsequent calculation of the TIV was performed with SPM12. We used the standard segmentation algorithm for automatic segmentation of the white and grey matter and CSF. The volumes of the three tissue classes were added to the TIV. The volume ratio of WML was calculated from the ratio of lesion volume to TIV. To get a better overview, the results were multiplied by 100, which gave the volume of WML as a percentage of the intracranial volume (WMRI).

### Ophthalmic assessment and OCT Imaging

All participants underwent a complete ophthalmic assessment including retinal and optic nerve head imaging using spectral domain OCT-imaging. The time interval between MRI and ophthalmic assessment was 9,5 months at median (range: 6 weeks to 20 months). Participants were imaged with non-dilated pupils, using the same OCT-device (Spectralis HRA + OCT, version 6.5.4.0, Heidelberg Engineering, Heidelberg, Germany). Reporting of OCT imaging followed the APOSTEL 2.0 guidelines^56^. OCT-imaging was performed by two trained operators (SML, CFG).

**Figure 2:**
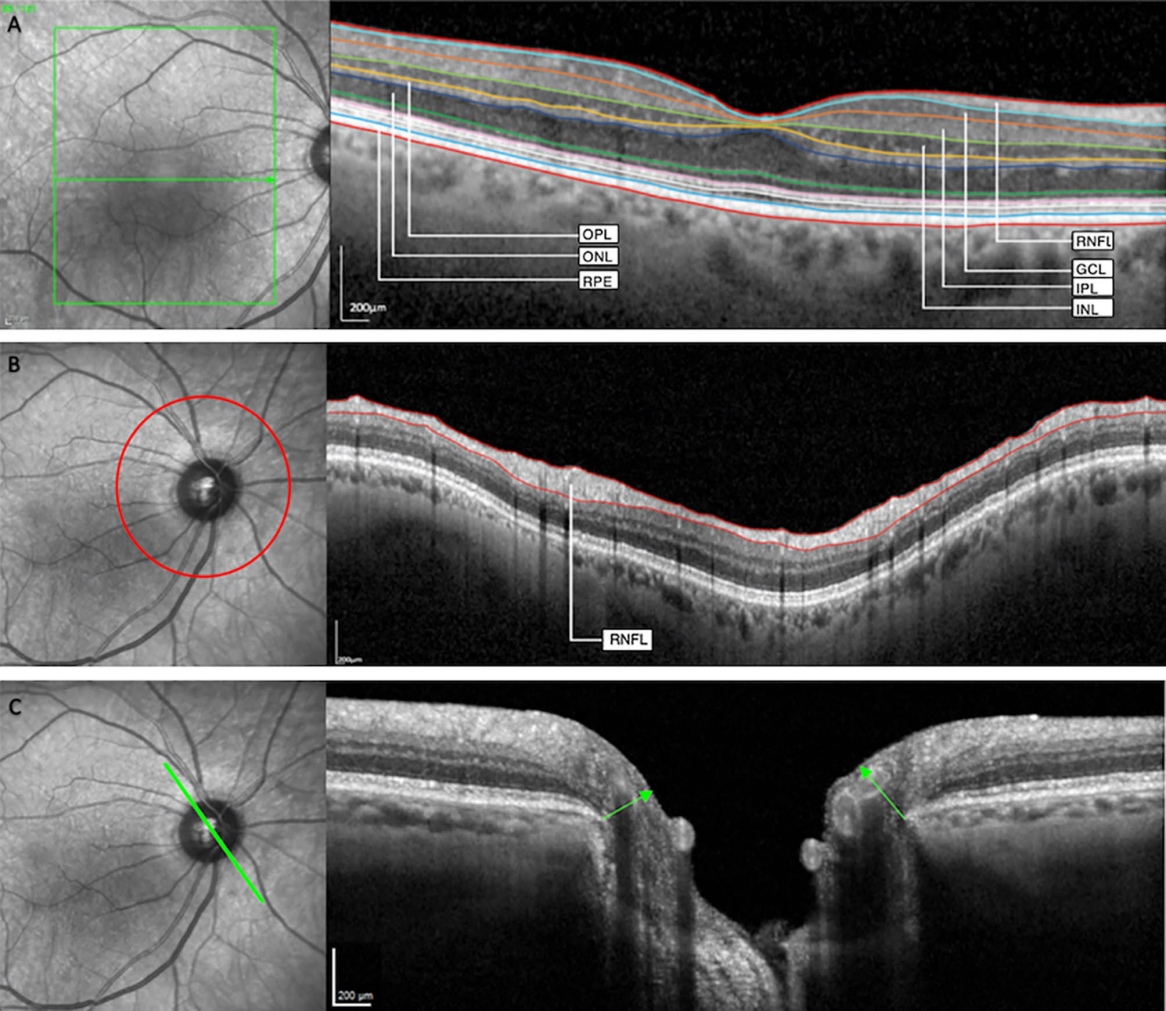
Optical coherence tomography-images of 75-year old participant. (**A**) Macular scan—different macular layers are segmented; (**B**) RNFL-scan—the peripapillary retinal nerve fiber layer (pRNFL) is segmented; (**C**) BMO-scan—the arrows show the Bruch’s membrane opening-minimum rim width (MRW). *RNFL* retinal nerve fiber layer, *GCL* ganglion cell layer, *IPL* inner plexiform layer, *INL* inner nuclear layer, *OPL* outer plexiform layer, *ONL* outer nuclear layer, *RPE* retinal pigment epithelium.

A macular volume scan (121 horizontal B-Scans, 25 frames, 20° × 25°), and a BMO scan (3.5 mm, 4.1 mm, and 4.7 mm diameter circular scans with 100 frames and automatic placement and 24 radial scans with 25 frames each) of the optic nerve head were acquired (Fig. 2). The integrated automatic real-time function adjusts for eye movement and offers a high resolution and reproducibility^57,58^. The BMO-scan combines the imaging of pRNFL (Fig. 2B) and the MRW (Fig. 2C). The MRW describes the anatomy of the optic nerve head and quantifies the minimum distance between the inner limiting membrane and the BMO (Fig. 2)^59^. It has been most widely used to capture pathological changes of the optic nerve head in e.g. glaucoma^41,60^.

The right eye of each patient was chosen for analysis except cases in whom any of the exclusion criteria applied for the right eye. In those cases, the left eye was chosen. OCT images with poor quality (i.e. < 20 dB single strength) or motion artifacts were excluded, as assessed by three graders (SML, JHT, CAT). Automated segmentation was performed, using the Heidelberg Eye Explorer (HEYEX 2, Heidelberg Engineering, Heidelberg, Germany). The automated segmentation boundaries of the individual layers (Fig. 2A), the pRNFL (Fig. 2B) and the MRW (Fig. 2C) were manually verified for accuracy and corrected if needed. The volumes were measured using the Early Treatment Diabetic Retinopathy Study grid^61^. We added the foveal and parafoveal volumes and used the 3 mm-circle-volumes as macular parameters. The outermost circle of the BMO scan (4.7 mm diameter) was used for pRNFL-analysis.

### Statistical analysis

Data analysis was performed with IBM SPSS Statistics (version 26.0). Descriptive statistics included mean scores which were compared using the Mann–Whitney-U-test for the overall cohort and for the age-matched sub-cohort (± 5 years).

Receiver operating characteristic (ROC) analysis with and without age adjustment was performed to determine the sensitivities of retinal parameters and MoCA score to detect CSVD (based on the Fazekas score) in our cohort. Because of a positive Pearson correlation coefficient between the OPL and the WMRI, the ROC-analysis for the OPL was performed with inversed OPL values. Binary logistic regression analysis was performed to determine the regression coefficients and the constant for age adjustment. Age and other significant parameters of the non-age-adjusted ROC analysis were used as independent variables in age-adjusted ROC analysis. Because of not normally-distributed WMRI residuals, WMRI values were logarithmically transformed for parametric statistical analyses.

In a subgroup analysis of all participants with CSVD, to whom none of the exclusion criteria applied, we investigated associations between MRI (WMRI, NOL) and OCT parameters using linear regression analysis. Multivariable regression models included parameters which were significantly associated in age-adjusted linear regressions and were calculated using backward elimination. If two dependent OCT-parameters correlated > 0.80 according to Pearson, only the one with a stronger correlation was included in the model to avoid multicollinearity.

The global statistical significance level was 0.05. This study followed STROBE checklist for observational research^62^.

## Supplementary Information

Supplementary Information.

## Data Availability

The data that support the findings of this study are included in the Supplement. Further data are available from the corresponding author upon reasonable request.

## Abbreviations

BMO: Bruch’s membrane opening
CADASIL: Cerebral autosomal dominant arteriopathy with subcortical infarcts and leukoencephalopathy
CSVD: Cerebral small vessel disease
FLAIR: Fluid-attenuated inversion recovery
GCL: Ganglion cell layer
INL: Inner nuclear layer
IPL: Inner plexiform layer
LPA: Lesion prediction algorithm
MoCA: Montreal cognitive assessment
MRW: Bruch’s membrane opening-minimum rim width
NOL: Number of white matter lesions
OCT: Optical coherence tomography
ONL: Outer nuclear layer
OPL: Outer plexiform layer
PMB: Papillo-macular bundle
pRNFL: Peripapillary retinal nerve fiber layer
ROC: Receiver operating characteristic
RPE: Retinal pigment epithelium
TIV: Total intracranial volume
WML: White matter lesions
WMRI: White matter lesions volume ratio index
