# Supplementary material for: Structural retinal changes in cerebral small vessel disease
Source: Sci Rep. 2022 Jun 3;12:9315. doi: 10.1038/s41598-022-13312-z (PMC9166694; doi:10.1038/s41598-022-13312-z)
Supplement: Supplementary file 1 — Supplementary Information. [file 41598_2022_13312_MOESM1_ESM.pdf]

Supplementary Material - Structural Retinal Changes in Cerebral Small Vessel Disease

S. Magdalena Langner[1+], Jan H. Terheyden MD[1+], Clara F. Geerling[1], Christine Kindler MD[2,3], Vera C. W. Keil MD[4], Christopher A. Turski MD[1,3], Gabrielle N. Turski MD[1,3], Charlotte Behning[5], Maximilian W. M. Wintergerst MD[1], Gabor C. Petzold MD[2,3,6#], Robert P. Finger MD, PhD[1#*]

[1] Department of Ophthalmology, University Hospital Bonn, Bonn, Germany
[2] Department of Neurology, University Hospital Bonn, Bonn, Germany
[3] German Center for Neurodegenerative Diseases (DZNE), Bonn, Germany
[4] Department of Radiology, Amsterdam UMC, location VUmc, De Boelelaan 1117, 1081 HV Amsterdam, The Netherlands
[5] Institute for Medical Biometry, Informatics and Epidemiology, University Hospital Bonn, Bonn, Germany
[6] Division of Vascular Neurology, University Hospital Bonn, Bonn, Germany

[+]These authors contributed equally.
[#]These authors jointly supervised the work.

Corresponding author:
Univ.-Prof. Dr. med. Robert Finger, PhD
Dpt. Ophthalmology, University of Bonn
Ernst-Abbe-Str. 2, 53127 Bonn, Germany
Email: robert.finger@ukbonn.de
Telephone: +49 228 287 155 05

Supplementary Table 1

Results of linear regression of MoCA, structural retinal parameters and white matter lesions volume ratio index in patients with cerebral small vessel disease with and without adjusting for age

	WMRI association		WMRI association, age adjusted	
	β [95% CI]	*p*	β [95% CI]	*p*
MoCA	-0.037 [-0.093, 0.020]	*0.192*	0.002 [-0.046, 0.050]	*0.940*
Total Retina				
total volume	-1.178 [-3.609, 1.254]	0.328	-0.465 [-2.365, 1.435]	0.618
Foveal	1.049 [-9.481, 11.579]	0.839	-1.099 [-9.135, 6.936]	0.780
pf. nasal	-6.334 [-17.468, 4.799]	0.252	-2.966 [-11.696, 5.765]	0.490
pf. inferior	-5.468 [-14.702, 3.766]	0.234	-1.959 [-9.320, 5.401]	0.588
pf. temporal	-4.615 [-16.010, 6.780]	0.412	-0.131 [-9.145, 8.884]	0.976
pf. superior	-9.035 [-21.006, 2.936]	0.133	-3.453 [-13.263, 6.357]	0.475
GCL				
total volume	-2.300 [-9.065, 4.465]	0.490	-2.317 [-7.401, 2.767]	0.356
foveal	-3.850 [-85.319, 77.619]	0.923	-27.013 [-88.617, 34.590]	0.374
pf. nasal	-2.336 [-30.499, 25.828]	0.866	-5.486 [-26.766, 15.793]	0.600
pf. inferior	-10.201 [-30.225, 9.823]	0.304	-8.973 [-24.021, 6.075]	0.230
pf. temporal	-8.748 [-36.888, 19.391]	0.528	-7.642 [-28.904, 13.620]	0.465
pf. superior	-12.709 [-47.05, 21.640]	0.453	-9.592 [-35.651, 16.468]	0.455
IPL				
total volume	-7.740 [-18.521, 3.042]	0.152	-4.809 [-13.210, 3.592]	0.249
foveal	19.902 [-30.689, 70.494]	0.425	13.605 [-24.923, 52.133]	0.473
pf. temporal	-12.151 [-60.064, 35.762]	0.606	-6.375 [-42.882, 30.133]	0.722
pf. superior	-16.579 [-62.269, 29.111]	0.462	-12.150 [-46.845, 22.545]	0.477
INL				
total volume	-3.418 [-16.879, 10.042]	0.606	-1.520 [-11.795, 8.755]	0.763
foveal	15.882 [-34.951, 66.714]	0.526	12.612 [-25.892, 51.116]	0.505
pf. nasal	-1.645 [-53.175, 49.886]	0.948	1.003 [-38.068, 40.074]	0.958
pf. inferior	-17.049 [-57.472, 23.374]	0.393	2.855 [-29.651, 35.360]	0.858
pf. temporal	4.247 [-47.259, 55.752]	0.867	-11.843 [-51.280, 27.595]	0.541
pf. superior	-41.327 [-89.970, 7.315]	0.092	-28.994 [-66.592, 8.603]	0.125
OPL				
total volume	5.550 [-2.830, 13.930]	0.185	0.286 [-6.812, 7.384]	0.934
pf. nasal	5.850 [-14.877, 26.576]	0.566	-5.563 [-22.081, 10.954]	0.494
pf. inferior	-0.695 [-21.559, 20.169]	0.946	-4.731 [-20.527, 11.065]	0.542
pf. temporal	28.158 [-16.559, 72.876]	0.207	14.020 [-21.180, 49.220]	0.419
pf. superior	21.455 [-2.487, 45.396]	0.077	6.562 [-14.170, 27.294]	0.520
ONL				
total volume	-3.075 [-8.247, 2.096]	0.232	1.218 [-3.309, 5.745]	0.584
foveal	-15.908 [-51.350, 19.534]	0.364	-6.488 [-34.037, 21.062]	0.631
pf. nasal	-8.656 [-23.050, 5.738]	0.227	2.763 [-9.761, 15.287]	0.653
pf. inferior	0.965 [-17.015, 18.946]	0.913	6.245 [-7.342, 19.832]	0.352
pf. temporal	-11.451 [-36.783, 13.881]	0.361	5.626 [-15.413, 26.665]	0.586

pf. superior	-16.610 [-35.134, 1.913]	0.077	1.451 [-16.421, 19.322]	0.868
RPE*				
total volume	9.465 [-6.978, 25.908]	0.247	8.363 [-3.960, 20.686]	0.174
pf. nasal	27.025 [-23.890, 77.940]	0.285	25.919 [-12.040, 63.878]	0.172
pf. inferior	17.319 [-38.307, 72.944]	0.527	17.962 [-23.858, 59.783]	0.384
pf. temporal	29.187 [-35.632, 94.006]	0.363	20.876 [-28.459, 70.210]	0.391
pf. superior	29.142 [-20.976, 79.260]	0.242	24.535 [-13.189, 62.259]	0.192
pRNFL				
total	-0.027 [-0.058, 0.004]	0.082	-0.020 [-0.044, 0.004]	0.095
nasal	-0.018 [-0.055, 0.018]	0.312	-0.008 [-0.037, 0.020]	0.554
N/T	0.892 [-0.494, 2.277]	0.197	0.893 [-0.136, 1.921]	0.086
nasal inferior	-0.011 [-0.029, 0.006]	0.198	-0.003 [-0.018, 0.011]	0.646
temporal inferior	0.001 [-0.015, 0.018]	0.862	-0.002 [-0.015, 0.011]	0.726
temporal superior	-0.013 [-0.030, 0.003]	0.102	-0.010 [-0.023, 0.002]	0.101
nasal superior	-0.019 [-0.037, 0.000]	0.052	-0.013 [-0.028, 0.002]	0.094
MRW				
total	-0.004 [-0.008, 0.000]	0.075	-0.002 [-0.006, 0.001]	0.233
N/T	0.205 [-0.636, 1.047]	0.619	0.006 [-0.649, 0.660]	0.986
temporal	-0.004 [-0.007, 0.000]	0.054	-0.002 [-0.005, 0.001]	0.239
temporal superior	-0.003 [-0.007, 0.001]	0.155	-0.001 [-0.004, 0.002]	0.389

Associations between retinal parameters and WMRI were assessed using linear regression. Model 1: univariate regression, Model 2: multivariable regression adjusted for age.

Note: The volumes of the macular layers correspond to the foveal and parafoveal volumes (3-mm-circle).

** The linear regression analysis of the foveal RPE volume is not listed, because the value contained a constant.*

Abbreviations: MoCA, Montreal Cognitive Assessment; WMRI, white matter lesions volume ratio index; β, standardized coefficient; CI, confidence interval; pf., parafoveal; GCL, ganglion cell layer; IPL, inner plexiform layer; INL, inner nuclear layer; OPL, outer plexiform layer; ONL, outer nuclear layer; RPE, retinal pigment epithelium; pRNFL, peripapillary retinal nerve fiber layer; N/T, ratio nasal to temporal; MRW, Bruch's membrane opening-minimum rim width.

Supplementary Table 2

Results of linear regression of MoCA, structural retinal parameters and number of white matter lesions in patients with cerebral small vessel disease

	NOL association		NOL association, age adjusted	
	β [95%-CI]	*p*	β [95%-CI]	*p*
MoCA	-0.568 [-1.322, 0.186]	0.133	-0.154 [-0.875, 0.567]	0.663
Total Retina				
total volume	-26.772 [-58.601, 5.057]	0.096	-18.997 [-46.636, 8.643]	0.169
Foveal	-41.798 [-183.956, 100.359]	0.550	-67.421 [-185.310, 50.468]	0.249
pf. nasal	-128.036 [-274.181, 18.108]	0.083	-91.154 [-218.363, 36.054]	0.152
pf. inferior	-99.660 [-222.101, 22.781]	0.106	-61.301 [-169.864, 47.262]	0.255
pf. temporal	-117.923 [-267.184, 31.338]	0.116	-70.104 [-202.668, 62.459]	0.286
GCL				
pf. inferior	-208.231 [-472.662, 56.200]	0.117	-194.188 [-413.160, 24.785]	0.080
pf. temporal	-268.182 [-637.470, 101.106]	0.147	-255.520 [-561.100, 50.060]	0.097
pf. superior	-343.939 [-794.266, 106.387]	0.128	-308.599 [-684.025, 66.826]	0.103
IPL				

total volume	-135.669 [-277.840, 6.502]	0.061	-103.199 [-225.920, 19.521]	0.095
foveal	-99.451 [-794.603, 595.702]	0.771	-174.010 [-756.264, 408.243]	0.543
pf. temporal	-140.777 [-792.741, 511.188]	0.660	-74.153 [-624.777, 476.472]	0.783
pf. superior	-336.607 [-948.791, 275.577]	0.268	-286.036 [-800.480, 228.408]	0.262
INL				
total volume	-49.425 [-232.181, 133.330]	0.582	-27.606 [-182.268, 127.055]	0.716
foveal	25.275 [-671.006, 721.555]	0.941	-12.679 [-598.248, 572.889]	0.965
pf. nasal	-46.667 [-746.619, 653.286]	0.892	-16.154 [-604.788, 572.479]	0.955
pf. inferior	-204.225 [-755.296, 346.845]	0.452	24.631 [-465.316, 514.578]	0.918
pf. temporal	108.333 [-590.460, 807.126]	0.752	-75.102 [-673.163, 522.959]	0.798
pf. superior	-481.667 [-1153.18, 189.853]	0.152	-339.539 [-917.692, 238.614]	0.237
OPL				
total volume	78.078 [-35.477, 191.633]	0.169	19.658 [-86.976, 126.293]	0.707
pf. nasal	151.913 [-124.605, 428.430]	0.269	29.830 [-221.204, 280.863]	0.808
pf. inferior	-95.355 [-376.130, 185.420]	0.491	-143.117 [-375.285, 89.050]	0.215
pf. temporal	205.804 [-416.134, 827.742]	0.502	38.041 [-499.455, 575.538]	0.885
ONL				
total volume	-59.169 [-127.286, 8.949]	0.086	-16.159 [-84.460, 52.141]	0.630
foveal	-214.674 [-696.341, 266.993]	0.367	-106.870 [-521.530, 307.789]	0.600
pf. nasal	-179.439 [-366.875, 7.998]	0.060	-67.853 [-255.187, 119.481]	0.462
pf. inferior	20.636 [-223.577, 264.849]	0.863	81.827 [-123.803, 287.457]	0.420
pf. temporal	-222.917 [-560.770, 114.936]	0.186	-41.896 [-360.383, 276.591]	0.788
RPE				
total volume	104.301 [-121.254, 329.856]	0.350	91.574 [-97.630, 280.777]	0.328
pf. nasal	298.864 [-398.482, 996.210]	0.386	286.103 [-296.642, 868.849]	0.321
pf. inferior	324.342 [-425.840, 1074.524]	0.382	331.774 [-293.084, 956.633]	0.284
pf. temporal	467.273 [-407.523, 1342.068]	0.282	372.219 [-366.307, 1110.745]	0.309
pf. superior	118.333 [-580.184, 816.851]	0.730	64.522 [-524.147, 653.192]	0.823
pRNFL*				
total	-0.386 [-0.805, 0.034]	0.070	-0.302 [-0.657, 0.052]	0.091
nasal	-0.132 [-0.644, 0.379]	0.598	-0.010 [-0.443, 0.424]	0.964
nasal inferior	-0.102 [-0.348, 0.145]	0.403	-0.002 [-0.219, 0.216]	0.989
temp. inferior	-0.066 [-0.297, 0.166]	0.562	-0.112 [-0.301, 0.078]	0.234
nasal superior	-0.258 [-0.516, 0.000]	0.050	-0.187 [-0.410, 0.036]	0.097
MRW				
N/T	2.890 [-8.706, 14.486]	0.612	0.480 [-9.318, 10.277]	0.920

Associations between retinal parameters and NOL were assessed using linear regression. Model 1: univariate regression, Model 2: multivariable regression adjusted for age.

Note: The volumes of the macular layers correspond to the foveal and parafoveal volumes (3-mm-circle).

** The linear regression analysis of the foveal RPE volume is not listed, because the value contained a constant.*

Abbreviations: MoCA, Montreal Cognitive Assessment; NOL, number of white matter lesions; β, standardized coefficient; CI, confidence interval; GCL, ganglion cell layer; IPL, inner plexiform layer; INL, inner nuclear layer; OPL, outer plexiform layer; ONL, outer nuclear layer; RPE, retinal pigment epithelium; pRNFL, peripapillary retinal nerve fiber layer; MRW, Bruch's membrane opening-minimum rim width; N/T, ratio nasal to temporal.

Supplementary Table 3

Results of structural retinal parameters in subjects with cerebral small vessel disease

Total retinal volume (mm^3)	
total	2.31 ± 0.11
foveal	0.22 ± 0.02
pf. nasal	0.53 ± 0.02
pf. inferior	0.52 ± 0.03
pf. temporal	0.51 ± 0.02
pf. superior	0.53 ± 0.02
GCL volume (mm^3)	
total	0.31 ± 0.04
foveal	0.01 ± 0.00
pf. nasal	0.08 ± 0.01
pf. inferior	0.08 ± 0.01
pf. temporal	0.07 ± 0.01
pf. superior	0.08 ± 0.01
IPL volume (mm^3)	
total	0.27 ± 0.02
foveal	0.01 ± 0.01
pf. nasal	0.06 ± 0.01
pf. inferior	0.06 ± 0.01
pf. temporal	0.06 ± 0.01
pf. superior	0.06 ± 0.01
INL volume (mm^3)	
total	0.27 ± 0.02
foveal	0.02 ± 0.01
pf. nasal	0.06 ± 0.01
pf. inferior	0.07 ± 0.01
pf. temporal	0.06 ± 0.01
pf. superior	0.06 ± 0.01
OPL volume (mm^3)	
total	0.21 ± 0.03
foveal	0.02 ± 0.00
pf. nasal	0.05 ± 0.01
pf. inferior	0.05 ± 0.01
pf. temporal	0.04 ± 0.01
pf. superior	0.05 ± 0.01
ONL volume (mm^3)	
total	0.52 ± 0.05
foveal	0.08 ± 0.01
pf. nasal	0.12 ± 0.02
pf. inferior	0.10 ± 0.01
pf. temporal	0.12 ± 0.01
pf. superior	0.11 ± 0.01
RPE volume (mm^3)	
total	0.10 ± 0.02
foveal	0.01 ± 0.00
pf. nasal	0.02 ± 0.01

pf. inferior	0.02 ± 0.00
pf. temporal	0.02 ± 0.00
pf. superior	0.02 ± 0.01
pRNFL thickness (µm)	
total	73.62 ± 8.21
nasal	58.69 ± 7.18
N/T	1.06 ± 0.19
nasal inferior	71.88 ± 14.75
temporal inferior	119.23 ± 15.84
temporal	56.69 ± 8.62
PMB	41.15 ± 6.71
temporal superior	110.58 ± 15.55
nasal superior	72.08 ± 13.19
BMO-MRW (µm)	
total	323.04 ± 59,37
nasal	361.92 ± 72.29
N/T	1.61 ± 0.32
nasal inferior	393.27 ± 74.21
temporal inferior	334.81 ± 67.48
Temporal	233.35 ± 65.52
PMB	216.69 ± 71.20
temporal superior	289.12 ± 66.75
nasal superior	339.08 ± 70.80

Values are mean ± SD. The volumes of the macular layers correspond to the foveal and parafoveal volumes (3-mm-circle)

Abbreviations: pf., parafoveal; GCL, ganglion cell layer; IPL, inner plexiform layer; INL, inner nuclear layer; OPL, outer plexiform layer; ONL, outer nuclear layer; RPE, retinal pigment epithelium; pRNFL, peripapillary retinal nerve fiber layer; N/T, ratio nasal to temporal; PMB, papillo-macular bundle; BMO-MRW, Bruch's membrane opening-minimum rim width.
